# Synthesis and crystal structure of (*E*)-*N*-[(2-bromo­phen­yl)methyl­idene]-3,5-bis­(tri­fluoro­meth­yl)aniline

**DOI:** 10.1107/S2056989025007650

**Published:** 2025-09-05

**Authors:** Nicholas B. Kingsley, Tyler J. Doyon, Daron E. Janzen

**Affiliations:** a Department of Green Chemistry and Biochemistry, University of Michigan-Flint, 303 E. Kearsley St, Flint, MI 48502, USA; b Department of Chemistry and Biochemistry, University of Wisconsin-Eau Claire, Phillips Science Hall, 101 Roosevelt Ave., Eau Claire, WI 54701, USA; c Dept of Chemistry & Biochemistry St. Catherine University, 2004 Randolph Avenue St Paul MN 55105 USA; Illinois State University, USA

**Keywords:** crystal structure, tri­fluoro­meth­yl, aryl imine ligands

## Abstract

The title compound C_15_H_8_BrF_6_N, was prepared by a condensation reaction of 2-bromo­benzaldehyde and 3,5-bis­(tri­fluoro­meth­yl)aniline in the presence of anhydrous magnesium sulfate. The compound readily crystallizes from a concentrated hexa­nes solution in high yield. The imine bond is nearly planar with the 2-bromo­phenyl group, while the *N*-(3,5-bis­(tri­fluoro­meth­yl)phenyl moiety is significantly twisted from the imine group [49.61 (5)°]. The crystal packing involves short inter­molecular C—H⋯Br contacts linking zigzag ribbons flanked by fluorous layers.

## Abstract 2

The synthesis and crystal structure of (*E*)-*N*-[(2-bromo­phen­yl)methyl­idene]-3,5-bis­(tri­fluoro­meth­yl)aniline are reported.

## 1. Chemical context

Aryl­imine-type ligands have increasingly been used as ancillary ligands for a variety of metal complexes. (Dostál *et al.*, 2011 ▸; Hejda *et al.*, 2012 ▸; Hejda *et al.*, 2013 ▸, 2017 ▸; Joseph *et al.*, 2018 ▸; Kingsley *et al.*, 2016 ▸; Kořenková *et al.*, 2016 ▸; Kremláček *et al.*, 2018 ▸; Mungwe *et al.*, 2011 ▸; Novák *et al.*, 2014 ▸, 2016 ▸; Šimon *et al.*, 2013 ▸; Urbanová *et al.*, 2013 ▸, 2014 ▸; Vrána *et al.*, 2013 ▸; Zhao *et al.*, 2010 ▸, 2011 ▸) Synthesis of these ligands is typically performed with alkyl-substituted anilines or alkyl amines reacting with 2-brombenzaldehyde. There is inter­est in modifying the electronic structure of the ligands but this has been limited to substitutions on the aromatic ring of the benzaldehyde starting material. (Chen *et al.*, 2004 ▸; Li *et al.*, 2019 ▸). Our group is inter­ested in developing aryl­imine ligands with electron-withdrawing groups on the aromatic ring of the *N*-imine portion of the ligand. Herein we report the synthesis and crystal structure of (*E*)-*N*-[(2-bromo­phen­yl)methyl­idene]-3,5-bis­(tri­fluoro­meth­yl)aniline (**I**).
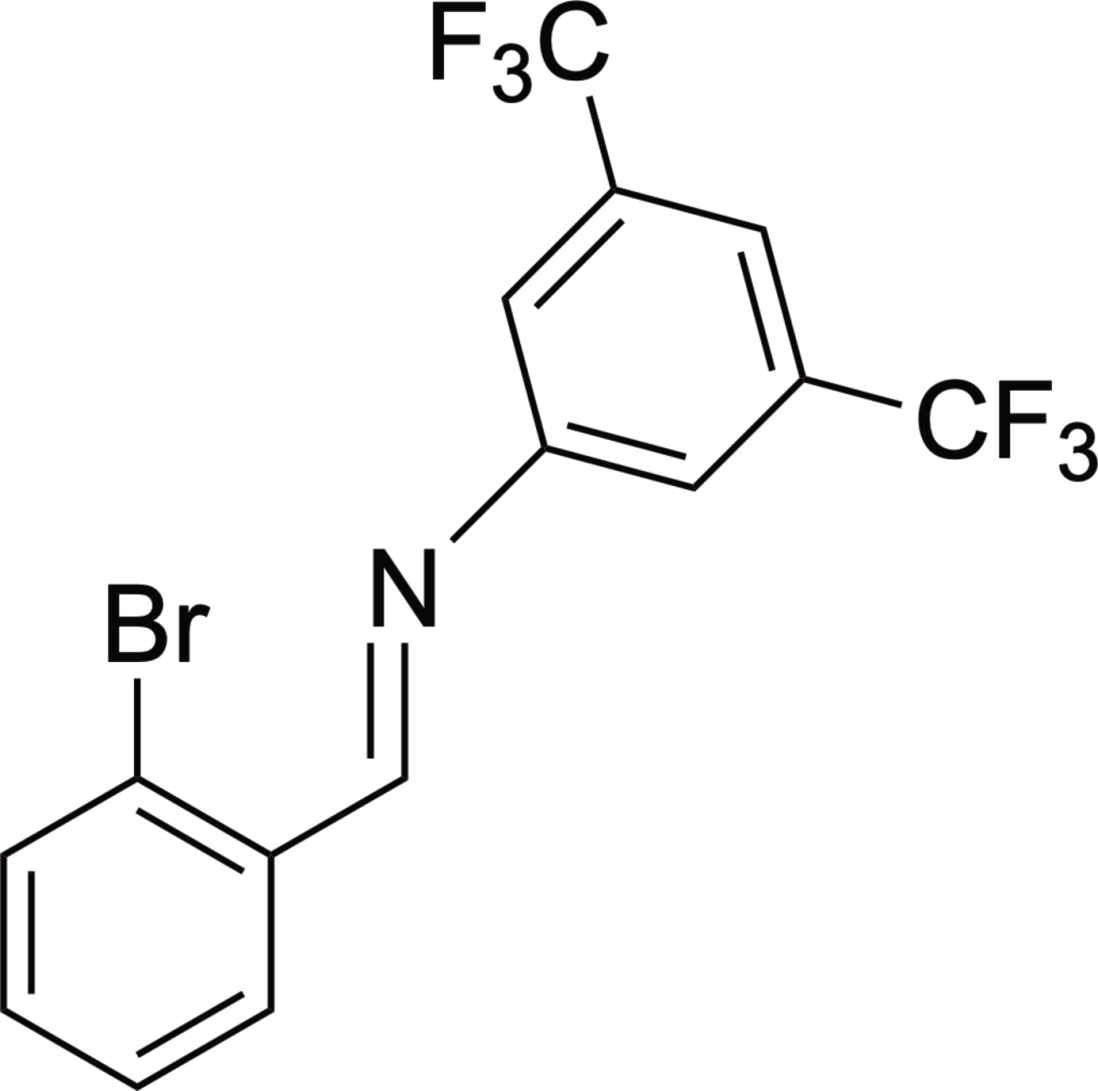


## 2. Structural commentary

**Figure 1:**
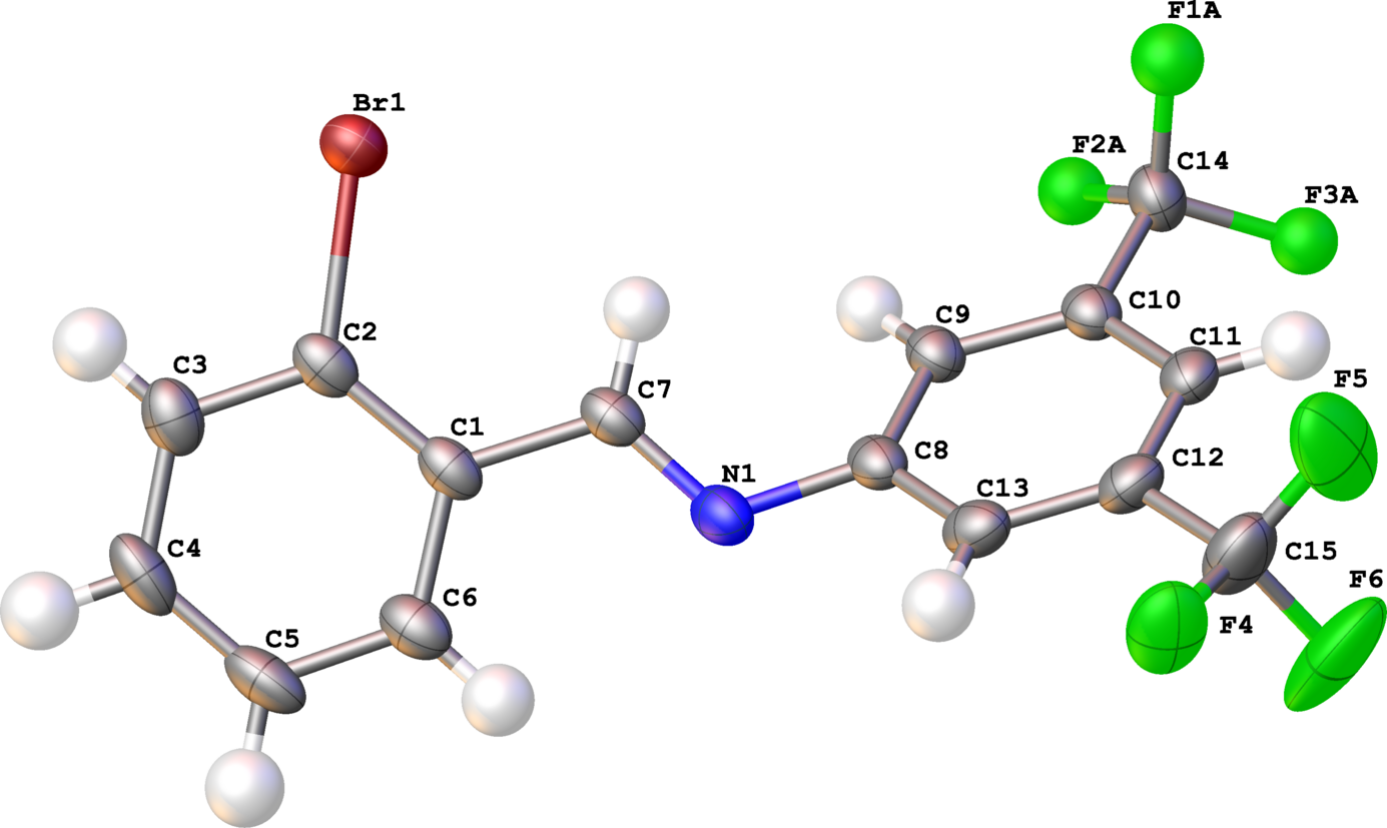
The mol­ecular structure of **I**. Only one of the three CF_3_ disorder model conformations shown. Thermal displacement ellipsoids are drawn at the 50% probability level.

A displacement ellipsoid plot of compound **I** is shown in Fig. 1 ▸. The imine bond length C7—N1 [1.280 (4) Å] and C7—N1—C8 bond angle [119.1 (2)°] are consistent with atom N1 being *sp*^2^ hybridized. The imine bond is oriented nearly coplanar with the C1–C6 phenyl ring [angle between least squares planes formed by C1–C6 and N1,C7,C1 = 5.9 (3)°]. Torsion angles (Table 1 ▸) near the imine bond demonstrate this as well as the twist of the C8–C13 phenyl ring relative to the imine bond plane formed by C8/N1/C7 is 42.0 (3)°). The angle between the least-squares planes formed by C1–C8/N1/Br1 and C8–C15/N1 of 49.61 (5)° is consistent with steric limitations influenced by atom positions of H7 and H9. Short intra­molecular inter­actions involving C—H bonds with acceptors are also present. A short C7—H7⋯Br1 contact (2.82 Å, length − vdW radii sums = −0.244 Å) and another contact involving C6—H6⋯N1 (2.52 Å, length − vdW sums = −0.342 Å) help define the observed conformation of **I** (Table 2 ▸).

## 3. Supra­molecular features

**Figure 2:**
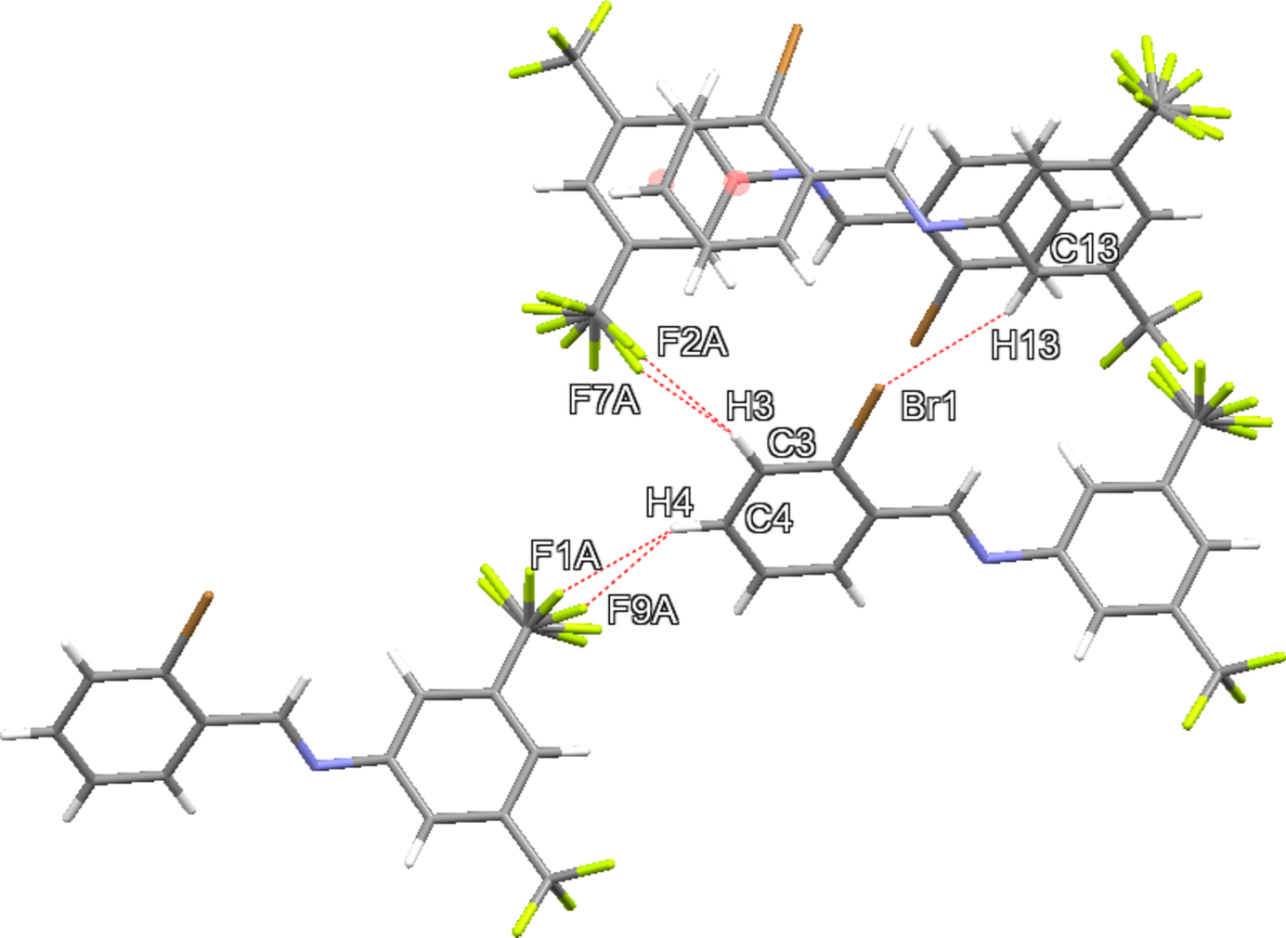
Inter­molecular inter­actions of **I** including centroids of the weak π–π dimer.

**Figure 3:**
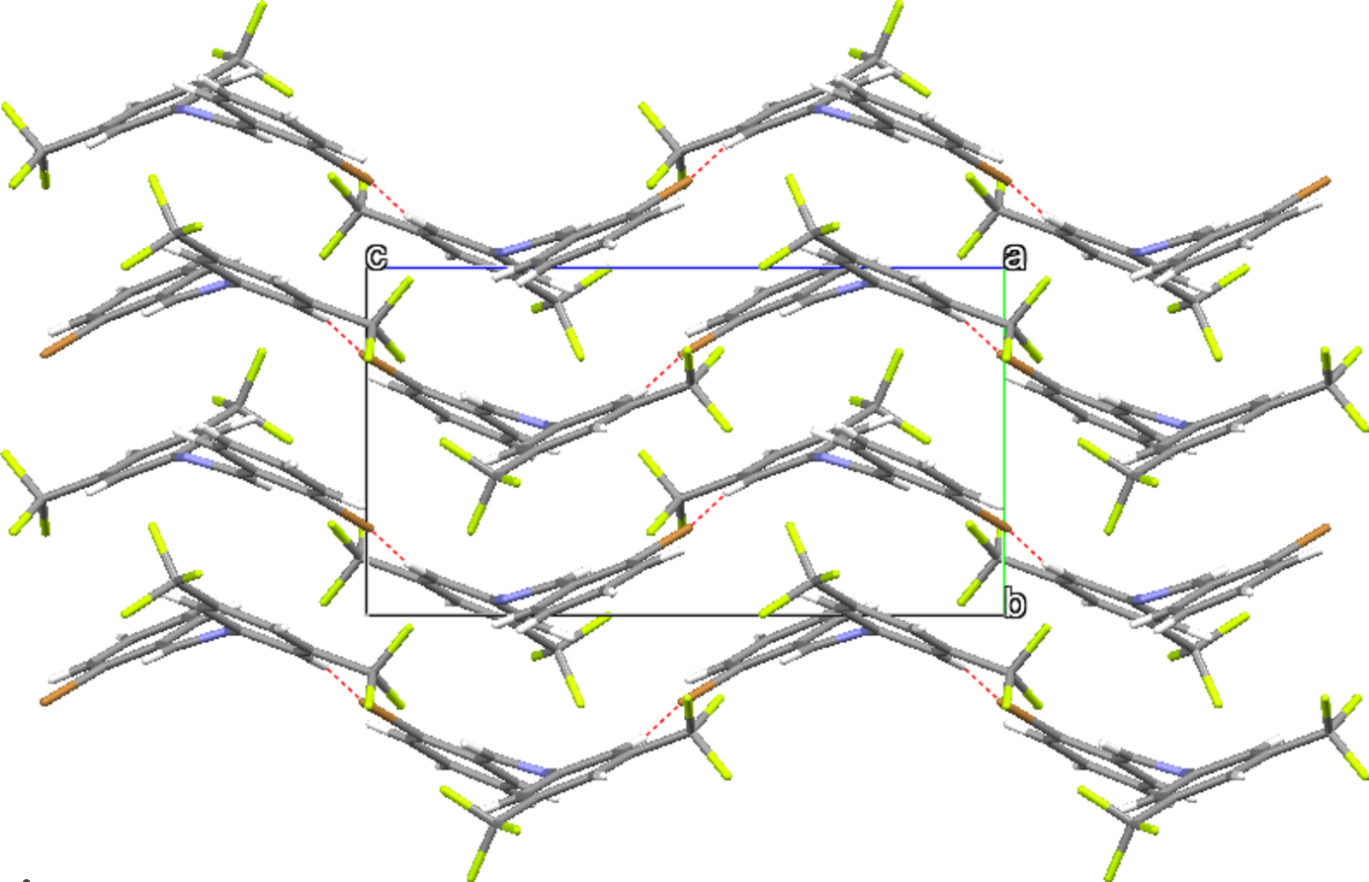
Packing diagram of **I** in a view along the *a*-axis direction. C—H⋯Br inter­actions are shown as dashed lines.

**Figure 4:**
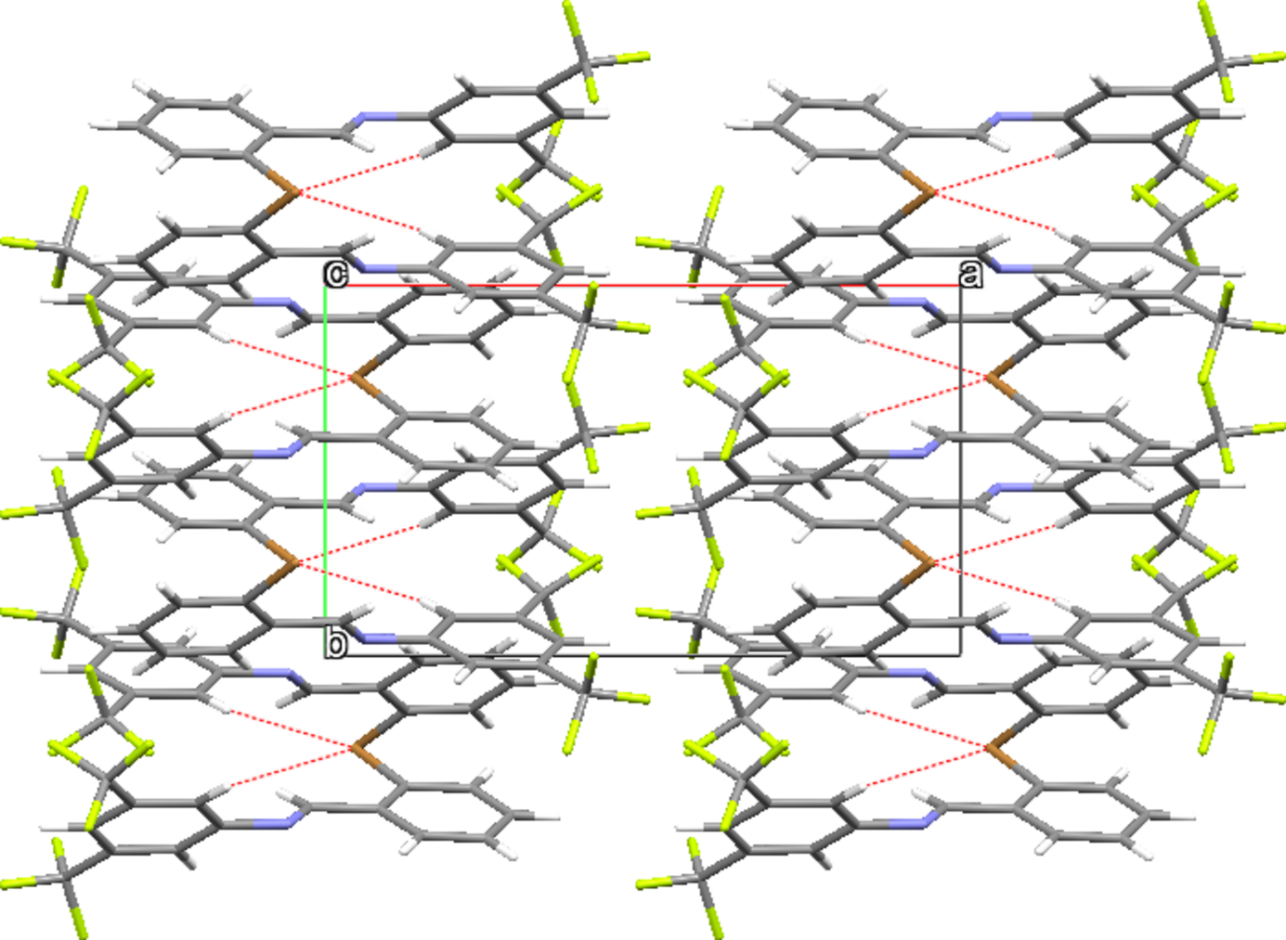
Packing diagram of **I** in a view along the *c*-axis direction. C—H⋯Br inter­actions are shown as dashed lines.

While no conventional hydrogen-bonding inter­actions are present, several short C—H⋯F contacts with fluorine atoms of the disordered CF_3_ group and hydrogen atoms H3 and H4 are present (Table 2 ▸, Fig. 2 ▸). A contact involving C13—H13⋯Br1 is also present, forming zigzag infinite ribbons of mol­ecules along (001) as shown in Fig. 3 ▸. The packing diagram of **I** (Fig. 4 ▸) demonstrates how the CF_3_ groups associate in fluorous layers in the *bc* plane at *x* = 0.5 with the ribbons of C—H⋯Br inter­actions between the CF_3_-rich layers. Weak π–π dimer inter­molecular inter­actions between a neighboring brominated phenyl ring (less electron poor) and the tri­fluoro­methyl­ated phenyl ring (more electron poor) are evident, with a 3.730 (2) Å centroid-to-centroid distance.

## 4. Database survey

A recent search in the CSD (version 6.00, last update April 2025; Groom *et al.*, 2016 ▸) using ConQuest (Bruno *et al.*, 2002 ▸) revealed the most closely related structures to **I** include a single *meta*-fluoro substitution (in place of a bis-tri­fluoro­methyl-substituted phenyl ring). The structure of 1-(2-bromo­phen­yl)-*N*-(3-fluoro­phen­yl)methanimine (FOBLUF; Kaur & Choudhury, 2014 ▸) demonstrates similar intra­molecular features to **I**, including the imine C—H⋯Br and phenyl *ortho* C—H⋯N distances (2.76 and 2.53 Å, respectively) as well as the twist angle between phenyl least-squares planes [40.97 (8)°]. Another related structure reported with a single electron-withdrawing *meta* substituent on the *N*-imine ring is *N*-(2-iodo­benzyl­idene)-3-nitro­aniline (MOPPOW; Wardell *et al.*, 2002 ▸). Similar to **I**, this mono-nitro-substituted structure also demonstrates analogous phenyl *ortho* C—H⋯N distances (2.58 Å) but the twist angle between phenyl least-squares planes (64.2°) is significantly different and may be related to the presence of the inter­molecular halogen bonding that is present (I⋯N distance = 3.487 Å, C8—I1⋯N1 = 160.1°). The structure of 2-({[3,5-bis­(tri­fluoro­meth­yl)phen­yl]imino}­meth­yl)-5-(di­methyl­amino)­phenol (BIDCUQ; Zhao *et al.*, 2023 ▸) possesses the same 3,5-bis­(tri­fluoro­meth­yl)phenyl moiety as **I**, but the imine-linked 2-hy­droxy phenyl group engages an intra­molecular inter­action (O1—H1⋯N2 = 1.84 Å), inducing a coplanar orientation of the phenyl rings. Also, the CF_3_ groups of BIDCUQ associate in fluorous layers (similar to **I**) between infinite single-distance π–π stacked aromatic regions (3.47 Å).

## 5. Synthesis and crystallization

2-Bromo­benzaldehyde (4.50 g, 24.3 mmol) was stirred in hexa­nes (250 mL) in the presence of anhydrous MgSO_4_ (1.0 g). 3,5-Bis(tri­fluoro­meth­yl)aniline (5.575 g, 24.3 mmol) was slowly added and the solution was allowed to stir for 2 h. MgSO_4_ was then removed via vacuum filtration and the filtrate cooled to 243 K for 48 h. The yellow precipitate was collected via vacuum filtration and purified through recrystallization from hexa­nes. The resulting material was light-yellow and crystalline. Yield: 7.62 g, 79%. ^1^H NMR (CDCl_3_, 400 MHz): δ 8.86 (*s*, 1H, C*H*=N), 8.22 (*dd*, ^3^*J*_HH_ = 7.7 Hz, ^4^*J*_HH_ = 1.9 Hz 1H, aryl H6), 7.76 (*br s*, 1H, aryl H11), 7.66 (*dd*, ^3^*J*_HH_ = 7.7 Hz, ^4^*J*_HH_ = 1.9 Hz 1H, aryl H3), 7.63 (*br s*, 2H, aryl H9, H13), 7.44 (*m*, 1H, aryl H5), 7.38 (*m*, 1H, aryl H4). ^13^C NMR {^1^H} (CDCl_3_, 100 MHz): δ 162.30 (*s*, C7), 153.15 (*s*, aryl C8), 133.81 (*s*, aryl C1), 133.62 (*s*, aryl C4), 133.58 (*s*, aryl C3), 132.79 (*q*, ^2^*J*_CF_ = 33.6 Hz, aryl C10, C12), 129.41 (*s*, aryl C6), 128.05 (*s*, aryl C5), 126.71 (*s*, aryl C2), 123.32 (*q*, ^1^*J*_CF_ = 272.8 Hz, C14, C15), 121.40 (*q*, ^3^*J*_CF_ = 2.7 Hz, aryl C9, C13), 119.71 (sept, ^3^*J*_CF_ = 3.8 Hz, aryl C11). M.p.: 361–363 K. X-ray quality crystals were grown from a concentrated solution in warm hexa­nes followed by slow cooling to room temperature and storage at 243 K for 96 h.

## 6. Refinement

Crystal data, data collection and structure refinement details are summarized in Table 3 ▸. Hydrogen atoms were refined with riding coordinates with fixed *U*_iso_(H) at 1.2 times of the riding C atom. Several restraints and constraints were used to model disorder in one of the CF_3_ groups. Distance restraints were employed for C14—F1*A*, C14—F2*A*, C14—F3*A*, C14—F4*A*, C14—F5*A*, C14—F6*A*, C14—F7*A*, C14—F8*A*, and C14—F9*A* with sigma of 0.02; F1*A*—F2*A*, F2*A*—F3*A*, F3*A*—F1*A*, F4*A*—F5*A*, F5*A*—F6*A*, F6*A*—F4*A*, F7*A*—F8*A*, F8*A*—F9*A*, and F9*A*—F7*A* with sigma of 0.04; and C10—F1*A*, C10—F2*A*, C10—F3*A*, C10—F4*A*, C10—F5*A*, C10—F6*A*, C10—F7*A*, C10—F8*A*, and C10—F9*A* with sigma of 0.1. *U*_aniso_ restraints were applied to C10, C14, F1*A*, F2*A*, F3*A*, F4*A*, F5*A*, F6*A*, F7*A*, F8*A*, and F9*A* within 2 Å with sigma of 0.04 and sigma for terminal atoms of 0.08 within 2 Å. Rigid body (RIGU) restraints were applied to C10, C14, F1*A*, F2*A*, F3*A*, F4*A*, F5*A*, F6*A*, F7*A*, F8*A*, F9*A* with sigma for 1–2 distances of 0.004 and sigma for 1–3 distances of 0.004. The three orientations of the CF_3_ group were fixed at sof values of 0.37 (F1*A*, F2*A*, F3*A*), 0.38 (F4*A*, F5*A*, F6*A*), and 0.25 (F7*A*, F8*A*, F9*A*).

## Supplementary Material

Crystal structure: contains datablock(s) I. DOI: 10.1107/S2056989025007650/ej2015sup1.cif

Structure factors: contains datablock(s) I. DOI: 10.1107/S2056989025007650/ej2015Isup2.hkl

Supporting information file. DOI: 10.1107/S2056989025007650/ej2015Isup3.cml

CCDC reference: 2483197

Additional supporting information:  crystallographic information; 3D view; checkCIF report

## Figures and Tables

**Table 1:** Selected torsion angles (°)

C2—C1—C7—N1	174.5 (2)	C7—N1—C8—C13	139.5 (3)
C6—C1—C7—N1	−5.9 (4)	C8—N1—C7—C1	176.7 (2)
C7—N1—C8—C9	−43.7 (4)		

**Table 2:** Hydrogen-bond geometry (Å, °)

*D*—H⋯*A*	*D*—H	H⋯*A*	*D*⋯*A*	*D*—H⋯*A*
C3—H3⋯F2*A*^i^	0.95	2.46	3.365 (7)	160
C3—H3⋯F7*A*^i^	0.95	2.25	3.063 (7)	143
C4—H4⋯F1*A*^ii^	0.95	2.54	3.411 (6)	153
C4—H4⋯F9*A*^ii^	0.95	2.36	3.152 (9)	140
C6—H6⋯N1	0.95	2.52	2.828 (4)	99
C7—H7⋯Br1	0.95	2.82	3.233 (3)	108
C13—H13⋯Br1^iii^	0.95	2.97	3.851 (3)	155

Symmetry codes: (i) 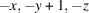; (ii) 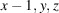; (iii) 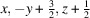.

**Table 3:** Experimental details

Crystal data	
Chemical formula	C_15_H_8_BrF_6_N
*M*_r_	396.13
Crystal system, space group	Monoclinic, *P*2_1_/*c*
Temperature (K)	100
*a*, *b*, *c* (Å)	13.261 (2), 7.7224 (12), 14.176 (2)
β (°)	93.394 (2)
*V* (Å^3^)	1449.2 (4)
*Z*	4
Radiation type	Mo *K*α
μ (mm^−1^)	2.90
Crystal size (mm)	0.25 × 0.20 × 0.15

Data collection	
Diffractometer	Bruker APEXII CCD
Absorption correction	Multi-scan (*SADABS*; Krause *et al.*, 2015 ▸)
*T*_min_, *T*_max_	0.575, 0.747
No. of measured, independent and observed [*I* > 2σ(*I*)] reflections	23866, 5193, 3166
*R*_int_	0.053
(sin θ/λ)_max_ (Å^−1^)	0.766

Refinement	
*R*[*F*^2^ > 2σ(*F*^2^)], *wR*(*F*^2^), *S*	0.046, 0.137, 1.06
No. of reflections	5193
No. of parameters	217
No. of restraints	189
H-atom treatment	H-atom parameters constrained
Δρ_max_, Δρ_min_ (e Å^−3^)	0.89, −0.68

Computer programs: *APEX2* and *SAINT* (Bruker 2017 ▸), *SHELXS97* (Sheldrick, 2008 ▸), *SHELXL2019/2* (Sheldrick, 2015 ▸), *OLEX2* (Dolomanov *et al.*, 2009 ▸), *Mercury* (Macrae *et al.*, 2020 ▸) and *publCIF* (Westrip, 2010 ▸).

## supplementary crystallographic information

### (E)-N-[(2-Bromophenyl)methylidene]-3,5-bis(trifluoromethyl)aniline Crystal data

**Table:**

C_15_H_8_BrF_6_N	*F*(000) = 776
*M**_r_* = 396.13	*D*_x_ = 1.816 Mg m^−^^3^
Monoclinic, *P*2_1_/*c*	Mo *K*α radiation, λ = 0.71073 Å
*a* = 13.261 (2) Å	Cell parameters from 6800 reflections
*b* = 7.7224 (12) Å	θ = 2.9–31.3°
*c* = 14.176 (2) Å	µ = 2.90 mm^−^^1^
β = 93.394 (2)°	*T* = 100 K
*V* = 1449.2 (4) Å^3^	Irregular brick, yellow
*Z* = 4	0.25 × 0.20 × 0.15 mm

### (E)-N-[(2-Bromophenyl)methylidene]-3,5-bis(trifluoromethyl)aniline Data collection

**Table:**

Bruker APEXII CCD diffractometer	3166 reflections with *I* > 2σ(*I*)
Radiation source: sealed tube	*R*_int_ = 0.053
φ and ω scans	θ_max_ = 33.0°, θ_min_ = 2.9°
Absorption correction: multi-scan (SADABS; Krause *et al.*, 2015)	*h* = −20→19
*T*_min_ = 0.575, *T*_max_ = 0.747	*k* = −11→11
23866 measured reflections	*l* = −21→21
5193 independent reflections	

### (E)-N-[(2-Bromophenyl)methylidene]-3,5-bis(trifluoromethyl)aniline Refinement

**Table:**

Refinement on *F*^2^	Primary atom site location: difference Fourier map
Least-squares matrix: full	Hydrogen site location: inferred from neighbouring sites
*R*[*F*^2^ > 2σ(*F*^2^)] = 0.046	H-atom parameters constrained
*wR*(*F*^2^) = 0.137	*w* = 1/[σ^2^(*F*_o_^2^) + (0.0521*P*)^2^ + 1.1524*P*] where *P* = (*F*_o_^2^ + 2*F*_c_^2^)/3
*S* = 1.06	(Δ/σ)_max_ = 0.001
5193 reflections	Δρ_max_ = 0.89 e Å^−^^3^
217 parameters	Δρ_min_ = −0.68 e Å^−^^3^
189 restraints	

### (E)-N-[(2-Bromophenyl)methylidene]-3,5-bis(trifluoromethyl)aniline Special details

**Table:**

Geometry. All esds (except the esd in the dihedral angle between two l.s. planes) are estimated using the full covariance matrix. The cell esds are taken into account individually in the estimation of esds in distances, angles and torsion angles; correlations between esds in cell parameters are only used when they are defined by crystal symmetry. An approximate (isotropic) treatment of cell esds is used for estimating esds involving l.s. planes.

### (E)-N-[(2-Bromophenyl)methylidene]-3,5-bis(trifluoromethyl)aniline Fractional atomic coordinates and isotropic or equivalent isotropic displacement parameters (Å^2^)

**Table:**

	*x*	*y*	*z*	*U*_iso_*/*U*_eq_	Occ. (<1)
Br1	−0.04663 (2)	0.75027 (4)	−0.00485 (2)	0.03627 (10)	
N1	0.05952 (17)	0.5457 (3)	0.28171 (18)	0.0335 (5)	
C1	−0.08432 (19)	0.5863 (3)	0.1738 (2)	0.0288 (5)	
C2	−0.12674 (19)	0.6484 (3)	0.08793 (19)	0.0293 (5)	
C3	−0.2302 (2)	0.6421 (4)	0.0656 (2)	0.0363 (6)	
H3	−0.257428	0.686307	0.006931	0.044*	
C4	−0.2931 (2)	0.5709 (4)	0.1295 (2)	0.0409 (7)	
H4	−0.363907	0.565656	0.114812	0.049*	
C5	−0.2530 (2)	0.5069 (4)	0.2152 (3)	0.0415 (7)	
H5	−0.296289	0.457257	0.258939	0.050*	
C6	−0.1504 (2)	0.5155 (3)	0.2369 (2)	0.0349 (6)	
H6	−0.123955	0.472455	0.296075	0.042*	
C7	0.02492 (19)	0.5917 (3)	0.1993 (2)	0.0289 (5)	
H7	0.070172	0.629651	0.154103	0.035*	
C8	0.16523 (19)	0.5438 (3)	0.3021 (2)	0.0297 (5)	
C9	0.23302 (19)	0.4776 (3)	0.2399 (2)	0.0288 (5)	
H9	0.209397	0.434980	0.179772	0.035*	
C10	0.33567 (19)	0.4746 (3)	0.26672 (19)	0.0290 (5)	
C11	0.3715 (2)	0.5378 (3)	0.35389 (19)	0.0317 (5)	
H11	0.441795	0.536850	0.371320	0.038*	
C12	0.3035 (2)	0.6022 (4)	0.41483 (19)	0.0346 (6)	
C13	0.2009 (2)	0.6037 (4)	0.3903 (2)	0.0350 (6)	
H13	0.154798	0.645660	0.433828	0.042*	
C14	0.4097 (2)	0.4018 (4)	0.2017 (2)	0.0355 (6)	
F1A	0.4543 (5)	0.5294 (7)	0.1579 (5)	0.0421 (12)*	0.37
F2A	0.3653 (4)	0.2903 (8)	0.1396 (4)	0.0366 (12)*	0.37
F3A	0.4837 (4)	0.3151 (8)	0.2533 (3)	0.0359 (11)*	0.37
F4A	0.4225 (4)	0.4969 (7)	0.1218 (4)	0.0469 (12)*	0.38
F5A	0.3808 (4)	0.2437 (6)	0.1641 (4)	0.0375 (11)*	0.38
F6A	0.5052 (4)	0.3815 (7)	0.2361 (4)	0.0438 (12)*	0.38
F7A	0.3694 (5)	0.3701 (10)	0.1145 (4)	0.0354 (14)*	0.25
F8A	0.4543 (7)	0.2606 (11)	0.2394 (6)	0.049 (2)*	0.25
F9A	0.4841 (7)	0.5119 (11)	0.1888 (7)	0.055 (2)*	0.25
C15	0.3438 (3)	0.6664 (6)	0.5095 (2)	0.0547 (9)	
F4	0.27725 (19)	0.7607 (3)	0.55397 (15)	0.0614 (6)	
F5	0.4281 (2)	0.7610 (4)	0.50321 (19)	0.0861 (10)	
F6	0.3718 (2)	0.5370 (4)	0.56638 (16)	0.0989 (10)	

### (E)-N-[(2-Bromophenyl)methylidene]-3,5-bis(trifluoromethyl)aniline Atomic displacement parameters (Å^2^)

**Table:**

	*U*^11^	*U*^22^	*U*^33^	*U*^12^	*U*^13^	*U*^23^
Br1	0.03181 (15)	0.04145 (16)	0.03619 (15)	0.00118 (12)	0.00736 (10)	0.00104 (12)
N1	0.0269 (11)	0.0333 (12)	0.0412 (12)	0.0038 (9)	0.0078 (9)	0.0063 (10)
C1	0.0238 (11)	0.0230 (11)	0.0405 (14)	−0.0009 (9)	0.0086 (10)	−0.0062 (10)
C2	0.0259 (12)	0.0251 (11)	0.0377 (14)	0.0000 (9)	0.0088 (10)	−0.0087 (10)
C3	0.0284 (13)	0.0351 (13)	0.0452 (16)	0.0030 (11)	0.0011 (11)	−0.0102 (12)
C4	0.0215 (12)	0.0402 (15)	0.062 (2)	−0.0021 (11)	0.0078 (12)	−0.0148 (14)
C5	0.0310 (15)	0.0332 (14)	0.063 (2)	−0.0032 (11)	0.0217 (14)	−0.0041 (14)
C6	0.0315 (14)	0.0265 (12)	0.0479 (16)	−0.0001 (10)	0.0120 (12)	−0.0021 (11)
C7	0.0257 (11)	0.0238 (11)	0.0380 (14)	−0.0006 (9)	0.0086 (10)	−0.0016 (10)
C8	0.0265 (12)	0.0274 (12)	0.0357 (13)	0.0010 (9)	0.0049 (10)	0.0085 (10)
C9	0.0253 (12)	0.0276 (12)	0.0339 (13)	−0.0014 (9)	0.0041 (10)	0.0035 (10)
C10	0.0269 (12)	0.0269 (11)	0.0336 (13)	−0.0008 (9)	0.0042 (10)	0.0052 (10)
C11	0.0309 (13)	0.0309 (13)	0.0329 (13)	−0.0003 (10)	−0.0005 (10)	0.0104 (10)
C12	0.0421 (15)	0.0353 (14)	0.0262 (12)	0.0044 (11)	0.0008 (11)	0.0072 (10)
C13	0.0380 (15)	0.0351 (14)	0.0324 (13)	0.0082 (11)	0.0074 (11)	0.0085 (11)
C14	0.0257 (12)	0.0401 (15)	0.0406 (15)	−0.0003 (10)	0.0004 (11)	0.0021 (12)
C15	0.059 (2)	0.072 (2)	0.0316 (16)	0.0147 (19)	−0.0048 (15)	0.0031 (16)
F4	0.0617 (14)	0.0846 (17)	0.0374 (10)	0.0156 (11)	−0.0001 (9)	−0.0144 (10)
F5	0.0564 (14)	0.144 (3)	0.0563 (15)	−0.0185 (15)	−0.0079 (11)	−0.0325 (15)
F6	0.139 (3)	0.119 (2)	0.0364 (12)	0.058 (2)	−0.0165 (14)	0.0149 (13)

### (E)-N-[(2-Bromophenyl)methylidene]-3,5-bis(trifluoromethyl)aniline Geometric parameters (Å, º)

**Table:**

Br1—C2	1.908 (3)	C10—C11	1.386 (4)
N1—C7	1.280 (4)	C10—C14	1.496 (4)
N1—C8	1.414 (3)	C11—H11	0.9500
C1—C2	1.396 (4)	C11—C12	1.378 (4)
C1—C6	1.400 (4)	C12—C13	1.384 (4)
C1—C7	1.473 (4)	C12—C15	1.499 (4)
C2—C3	1.391 (4)	C13—H13	0.9500
C3—H3	0.9500	C14—F1A	1.323 (6)
C3—C4	1.382 (4)	C14—F2A	1.342 (5)
C4—H4	0.9500	C14—F3A	1.364 (5)
C4—C5	1.388 (5)	C14—F4A	1.369 (5)
C5—H5	0.9500	C14—F5A	1.377 (5)
C5—C6	1.379 (4)	C14—F6A	1.338 (5)
C6—H6	0.9500	C14—F7A	1.339 (6)
C7—H7	0.9500	C14—F8A	1.336 (7)
C8—C9	1.392 (4)	C14—F9A	1.323 (8)
C8—C13	1.390 (4)	C15—F4	1.331 (4)
C9—H9	0.9500	C15—F5	1.343 (5)
C9—C10	1.392 (3)	C15—F6	1.324 (4)
			
C7—N1—C8	119.1 (2)	C12—C11—C10	118.9 (3)
C2—C1—C6	117.2 (2)	C12—C11—H11	120.5
C2—C1—C7	122.9 (2)	C11—C12—C13	121.1 (3)
C6—C1—C7	119.9 (3)	C11—C12—C15	117.9 (3)
C1—C2—Br1	122.07 (19)	C13—C12—C15	121.0 (3)
C3—C2—Br1	116.1 (2)	C8—C13—H13	120.0
C3—C2—C1	121.8 (3)	C12—C13—C8	120.0 (3)
C2—C3—H3	120.3	C12—C13—H13	120.0
C4—C3—C2	119.4 (3)	F1A—C14—C10	109.7 (3)
C4—C3—H3	120.3	F1A—C14—F2A	111.2 (4)
C3—C4—H4	120.0	F1A—C14—F3A	106.9 (4)
C3—C4—C5	120.1 (3)	F2A—C14—C10	111.5 (3)
C5—C4—H4	120.0	F2A—C14—F3A	108.1 (4)
C4—C5—H5	120.0	F3A—C14—C10	109.3 (3)
C6—C5—C4	120.0 (3)	F4A—C14—C10	115.2 (3)
C6—C5—H5	120.0	F4A—C14—F5A	101.6 (4)
C1—C6—H6	119.2	F5A—C14—C10	113.2 (3)
C5—C6—C1	121.6 (3)	F6A—C14—C10	117.4 (3)
C5—C6—H6	119.2	F6A—C14—F4A	101.7 (4)
N1—C7—C1	120.7 (2)	F6A—C14—F5A	105.9 (4)
N1—C7—H7	119.7	F7A—C14—C10	113.4 (3)
C1—C7—H7	119.7	F8A—C14—C10	110.5 (4)
C9—C8—N1	123.0 (3)	F8A—C14—F7A	111.3 (5)
C13—C8—N1	117.4 (2)	F9A—C14—C10	111.4 (4)
C13—C8—C9	119.6 (2)	F9A—C14—F7A	104.5 (6)
C8—C9—H9	120.3	F9A—C14—F8A	105.3 (6)
C10—C9—C8	119.4 (3)	F4—C15—C12	113.2 (3)
C10—C9—H9	120.3	F4—C15—F5	108.2 (3)
C9—C10—C14	120.4 (2)	F5—C15—C12	112.0 (3)
C11—C10—C9	121.0 (2)	F6—C15—C12	111.6 (3)
C11—C10—C14	118.6 (2)	F6—C15—F4	107.1 (3)
C10—C11—H11	120.6	F6—C15—F5	104.3 (3)
			
Br1—C2—C3—C4	−179.7 (2)	C9—C10—C14—F5A	−47.6 (4)
N1—C8—C9—C10	−177.6 (2)	C9—C10—C14—F6A	−171.5 (4)
N1—C8—C13—C12	178.9 (2)	C9—C10—C14—F7A	10.2 (5)
C1—C2—C3—C4	−0.6 (4)	C9—C10—C14—F8A	−115.5 (5)
C2—C1—C6—C5	0.2 (4)	C9—C10—C14—F9A	127.7 (5)
C2—C1—C7—N1	174.5 (2)	C10—C11—C12—C13	0.3 (4)
C2—C3—C4—C5	0.2 (4)	C10—C11—C12—C15	178.7 (3)
C3—C4—C5—C6	0.4 (4)	C11—C10—C14—F1A	−79.9 (4)
C4—C5—C6—C1	−0.7 (4)	C11—C10—C14—F2A	156.5 (4)
C6—C1—C2—Br1	179.45 (19)	C11—C10—C14—F3A	37.0 (4)
C6—C1—C2—C3	0.4 (4)	C11—C10—C14—F4A	−111.5 (4)
C6—C1—C7—N1	−5.9 (4)	C11—C10—C14—F5A	132.2 (3)
C7—N1—C8—C9	−43.7 (4)	C11—C10—C14—F6A	8.3 (5)
C7—N1—C8—C13	139.5 (3)	C11—C10—C14—F7A	−170.0 (4)
C7—C1—C2—Br1	−0.9 (3)	C11—C10—C14—F8A	64.3 (5)
C7—C1—C2—C3	−179.9 (2)	C11—C10—C14—F9A	−52.5 (6)
C7—C1—C6—C5	−179.5 (3)	C11—C12—C13—C8	−1.7 (4)
C8—N1—C7—C1	176.7 (2)	C11—C12—C15—F4	165.6 (3)
C8—C9—C10—C11	−0.6 (4)	C11—C12—C15—F5	43.0 (4)
C8—C9—C10—C14	179.1 (2)	C11—C12—C15—F6	−73.5 (4)
C9—C8—C13—C12	2.0 (4)	C13—C8—C9—C10	−0.8 (4)
C9—C10—C11—C12	0.9 (4)	C13—C12—C15—F4	−16.0 (5)
C9—C10—C14—F1A	100.3 (4)	C13—C12—C15—F5	−138.6 (3)
C9—C10—C14—F2A	−23.2 (5)	C13—C12—C15—F6	104.9 (4)
C9—C10—C14—F3A	−142.7 (4)	C14—C10—C11—C12	−178.9 (2)
C9—C10—C14—F4A	68.7 (4)	C15—C12—C13—C8	179.9 (3)

### (E)-N-[(2-Bromophenyl)methylidene]-3,5-bis(trifluoromethyl)aniline Hydrogen-bond geometry (Å, º)

**Table:**

*D*—H···*A*	*D*—H	H···*A*	*D*···*A*	*D*—H···*A*
C3—H3···F2*A*^i^	0.95	2.46	3.365 (7)	160
C3—H3···F7*A*^i^	0.95	2.25	3.063 (7)	143
C4—H4···F1*A*^ii^	0.95	2.54	3.411 (6)	153
C4—H4···F9*A*^ii^	0.95	2.36	3.152 (9)	140
C6—H6···N1	0.95	2.52	2.828 (4)	99
C7—H7···Br1	0.95	2.82	3.233 (3)	108
C13—H13···Br1^iii^	0.95	2.97	3.851 (3)	155

Symmetry codes: (i) −*x*, −*y*+1, −*z*; (ii) *x*−1, *y*, *z*; (iii) *x*, −*y*+3/2, *z*+1/2.
